# Factors linked to severe thrombocytopenia during antiviral therapy in patients with chronic hepatitis c and pretreatment low platelet counts

**DOI:** 10.1186/1471-230X-12-7

**Published:** 2012-01-18

**Authors:** Kung-Hung Lin, Ping-I Hsu, Hsien-Chung Yu, Chun-Ku Lin, Wei-Lun Tsai, Wen-Chi Chen, Hoi-Hung Chan, Kwok-Hung Lai

**Affiliations:** 1 Division of Gastroenterology, Department of Internal Medicine, Kaohsiung Veterans General Hospital, Kaohsiung, Taiwan; 2 Division of Internal Medicine, Kaohsiung Veterans General Hospital, Pingtung Branch, Taiwan; 3 Department of General Medicine, School of Medicine, College of Medicine, Taipei Medical University, Taipei, Taiwan

**Keywords:** HCV, peg-IFN-α, ribavirin, thrombocytopenia

## Abstract

**Background:**

Baseline low platelet count (< 150,000/μL) increases the risk of on-treatment severe thrombocytopenia (platelet count < 50,000/μL) in patients with chronic hepatitis C (CHC) undergoing antiviral therapy, which may interrupt treatment. The purpose of this study was to identify risk factors for severe thrombocytopenia during treatment for CHC in patients with baseline thrombocytopenia.

**Methods:**

Medical records were reviewed for 125 patients with CHC treated with antiviral therapy according to the standard of care, with regular follow-up examinations. Early platelet decline was defined as platelet decrease during the first 2 weeks of therapy.

**Results:**

Severe thrombocytopenia developed in 12.8% of patients with baseline thrombocytopenia, and predicted a higher therapeutic dropout rate. Multivariate analysis revealed baseline platelet count < 100,000/μL and rapid early platelet decline (> 30% decline in the first 2 weeks) were significantly associated with severe thrombocytopenia (*P *< 0.001 and 0.003, odds ratios, 179.22 and 45.74, respectively). In these patients, baseline PLT ≥ 100,000/μL and lack of rapid early platelet decline predicted absence of severe thrombocytopenia (negative predictive values were 95.1% and 96.6%, respectively). In contrast, baseline platelet count < 100,000/μL combined with rapid early platelet decline predicted severe thrombocytopenia (positive predictive value was 100%).

**Conclusions:**

For patients with CHC on antiviral therapy, baseline platelet counts < 100,000/μL and rapid early platelet decline can identify patients at high risk of developing on-treatment severe thrombocytopenia.

## Background

Patients with chronic hepatitis C (CHC) treated with antiviral therapy consisting of pegylated interferon-α (peg-IFN-α) and ribavirin experience a response superior to that of therapies used in the past. This combination is the current standard of care [1]; however, side effects, especially hematologic abnormalities, may decrease both therapeutic adherence and the therapeutic success rate. Thrombocytopenia is one of the potential hematologic abnormalities associated with peg-IFN-α-based therapy [2-4].

One recent study reported that development of a platelet count < 50,000/μL was independently associated with bleeding during antiviral therapy [5]. In clinical practice, there is no approved therapy for reversing the decline in platelet count, even though some anti-thrombocytopenia therapies are currently under investigation [6]. Reduction of the dose of peg-IFN-α (either peg-IFN-α-2a or peg-IFN-α-2b) is recommended for patients suffering from platelet counts < 50,000/μL, and cessation of anti-viral treatment is recommended for platelet counts < 25,000/μL [7]. Discontinuation of anti-viral therapy is the only way to prevent progressive thrombocytopenia; however, discontinuation of therapy may reduce the rate of viral clearance and sustained virological response (SVR) [8].

Studies to identify risk factors for antiviral-therapy-induced severe thrombocytopenia are essential, but are rarely conducted. Baseline thrombocytopenia is reportedly independently and strongly associated with the development of a platelet count < 50,000/μL during antiviral therapy [5]. Hence, this study was designed to document more clearly the changes in platelet counts, and to investigate risk factors for treatment-induced severe thrombocytopenia (i.e., platelet count < 50,000/μL) in patients with pretreatment thrombocytopenia, undergoing standard antiviral therapy for CHC.

## Methods

### Patients

Medical records were reviewed retrospectively for chronic hepatitis C virus (HCV)-infected patients who received peg-IFN-α and ribavirin combination therapy in Kaohsiung Veterans General Hospital, Taiwan between September 2003 and October 2010. All patients were seropositive for anti-HCV antibody (Ax SYM HCV 3.0; Abbott Laboratories, Wiesbaden-Delkenheim, Germany), and had detectable HCV-RNA (Light Cycler, Roche, Branchburg, NJ, USA), and alanine aminotransferase (ALT) levels above the upper limit of normal (ULN). Exclusion criteria included hepatic decompensation, concomitant hepatitis B virus or human immunodeficiency virus infection, hepatitis other than hepatitis C (e.g., autoimmune or alcoholic hepatitis), or a contraindication to treatment with IFN-α or ribavirin. Patients with a pretreatment normal platelet count were not included in the analysis. This study was approved by the Institutional Review Board (IRB) of the Kaohsiung Veterans General Hospital (the IRB number was VGHKS12-CT1-03).

### Treatment and Follow-up

All patients received combination therapy consisting of ribavirin and either peg-IFN-α-2a or peg-IFN-α-2b based on the standard of care protocol at the time of their treatment. For patients with genotype non-1, the duration of treatment was 24 weeks. For patients with genotype 1, the duration of treatment was 24 to 48 weeks, based on the rapid virologic response (RVR) after 4 weeks of treatment. The choice of peg-IFN-α-2a or peg-IFN-α-2b was not randomized, but was made at the discretion of the treating physician. Peg-IFN-α-2a was administered subcutaneously at a dose of 180 μg *q *7 days, whereas peg-IFN-α-2b was administered subcutaneously at a dose of 1.5 μg/kg body weight *q *7 days. Ribavirin was prescribed initially at a total daily dose of 1,000 mg for patients weighing ≤ 75 kg and 1,200 mg for patients weighing > 75 kg.

Baseline complete blood counts and differential counts, liver function tests, HCV-RNA, genotypes (by a 5' non-coding region- and core-based reverse transcriptase PCR assay with sequencing [9]), and sonographic results were obtained. Histologic evaluation (scoring by the METAVIR system [10]) was available in some patients, in whom liver biopsy was performed. Splenomegaly was defined as spleen with a long axis > 12 cm by ultrasound [11]. Cirrhosis was diagnosed by ultrasound (using standard criteria, including: coarse echotexture, irregular surface, blunt edge, hypertrophic left lobe, splenomegaly), endoscopy (esophageal or gastric varices), or liver biopsy. Thrombocytopenia was defined as platelet count < 150,000/μL. This was further classified as mild (platelet count ≥ 100,000/μL), moderate (50,000/μL-100,000/μL), and severe (< 50,000/μL) thrombocytopenia. Dangerous thrombocytopenia was defined as a platelet count < 25,000/μL. The rate of platelet decline was defined as the rate of decrease from baseline. After 24-week follow-up, a platelet count increasing from baseline moderate thrombocytopenia to mild, or with resolution from baseline thrombocytopenia to platelet count ≥ 150,000/μL, was categorized as improvement. In contrast, a platelet count decreasing from baseline mild or moderate thrombocytopenia to moderate or severe thrombocytopenia was categorized as deterioration. Stable platelet counts were categorized as stationary.

Information was obtained regarding adherence to treatment, clinical, neuropsychiatric, and hematologic adverse effects, and biochemical and virologic response, at 2-week intervals during the first month, 4-week intervals during the remaining treatment period, and at weeks 4, 12, and 24 after completion of treatment. During treatment, if significant hematologic side effects occurred (e.g. absolute neutrophil count [ANC] < 750/mm^3 ^or platelet count < 50,000/μL), the dose of peg-IFN-α-2a was decreased to 135 μg/week and the dose of peg-IFN-α-2b was decreased to 0.75 μg/kg/wk. For an ANC < 500/mm^3 ^or a platelet count < 25,000/μL, both peg-IFN-α-2a and peg-IFN- α-2b were discontinued. The dose of ribavirin was decreased by 200 mg/day if the hemoglobin (Hgb) declined to < 10 g/dL, and held if Hgb < 8.5 g/dL. If the adverse effects resolved or diminished, a return to initial dosing levels was permitted.

### Statistical Analysis

Quantitative parameters were expressed as median and 25^th ^- 75^th ^percentile values or mean ± standard deviation. Categorical variables were compared with a χ^2 ^test and Fisher exact test. The continuous variables were examined by Kolmogorov-Smirnov test first for their normality of distribution, and compared with a Student's t-test for data with normal distribution, or with a Mann-Whitney U-test for data without normal distribution. A Receiver Operating Characteristic (ROC) analysis was performed to identify the cut-off value with best accuracy, of continuous variables with *P *< 0.1. For identification of factors related to severe thrombocytopenia during combination therapy, multivariate logistic regression analysis was applied. All statistical analyses were based on two-side hypothesis tests with a significance level of *P *< 0.05. All data analyses were performed using SPSS for Windows (version 12; SPSS Inc., Chicago, IL, USA).

## Results

**Figure 1:**
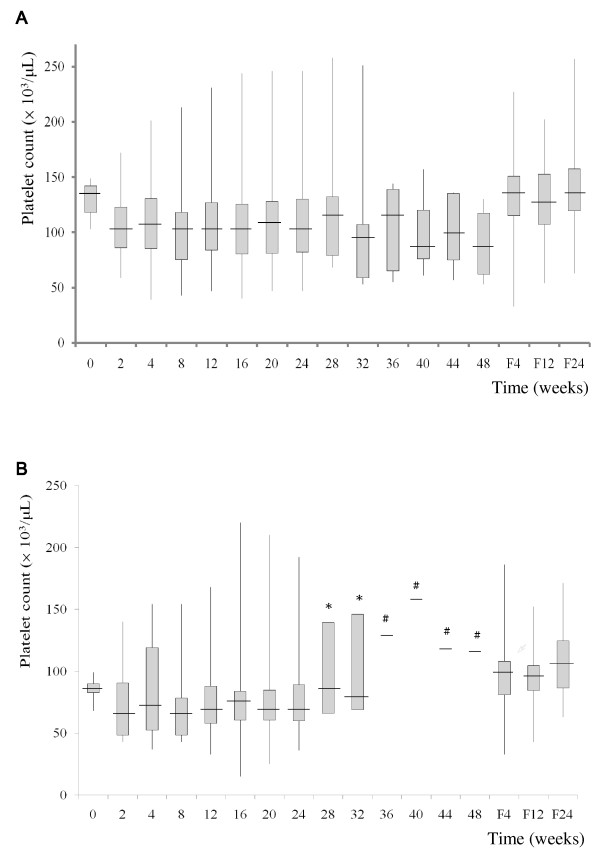
**Sequential changes in platelet counts during treatment with peginterferon-α and ribavirin in patients with pretreatment platelet counts between 100,000/μL and 150,000/μL (1A), and < 100,000/μL (1B)**. Each vertical line represents the range of platelet counts. Each shaded area represents the 25^th ^- 75^th ^percentiles, and each short horizontal line represents the median. F4, F12, and F24 mean follow-up weeks 4, weeks 12, and weeks 24, respectively. *: Number of the patients was 3. #: Number of the patients was 1.

In total, 125 patients with CHC and baseline thrombocytopenia who received antiviral therapy with peg-IFN-α and ribavirin were included in this study (Table 1). The prevalence of cirrhosis in patients with baseline thrombocytopenia was 22.4% (n = 28), and that of splenomegaly was 21.6% (n = 27). The changes in platelet counts are shown in Figure 1, divided into patients with pretreatment platelet counts between 100,000/μL and 150,000/μL (Figure 1A), and those with platelet count < 100,000/μL (Figure 1B). During antiviral therapy, platelet counts decreased rapidly at the end of the second week in both subgroups. Overall, 38 (30.4%), 25 (20%), 25 (20%), and 37 (29.6%) patients had a decline in platelet count of ≤ 10%, 10%-20%, 20%-30%, and > 30%, respectively. The lowest platelet counts were documented at a median time of 12 weeks after initiation of therapy. Platelet counts recovered after discontinuation of therapy.

**Table 1:** Characteristics of 125 patients with chronic hepatitis C

Variables	**Median (25**^**th**^**-75**^**th**^**percentile values) or number (percentage)**
Age (years)	57 (25-64)
Gender (male/female)	65/60 (52/48)
BMI (kg/m^2^)	24.8 (23.0-27.0)
Albumin (g/dL)	4.1 (3.9-4.4)
Total bilirubin (mg/dL)	0.7 (0.6-1.0)
AST (U/L)	107 (72-159)
ALT (U/L)	160 (109-259)
ALK-p (U/L)	96 (72-121)
PT INR	1.04 (1.01-1.08)
WBC (/mm^3^)	5,120 (4,403-6,083)
Hgb (g/dL)	14.4 (13.3-15.5)
Platelets (/μL)	125,000 (110,000-140,500)
Fibrosis (F1/2/3/4)	2/31/39/19 (1.6/24.8/31.2/15.2)^**#**^
Necroinflammatory activity (A0/1/2/3)	13/49/21/3 (10.4/38.2/16.8/2.4)^**#**^
Cirrhosis status (yes/no)	28/97 (22.4/77.6)
HCV-RNA (log_10_, IU/mL)	6.12 (5.39-6.66)
HCV genotype (1/2)	71/54 (56.8/43.2)
Splenomegaly (yes/no)	27/97 (21.6/77.6)^**§**^
Peg-IFN-α-2a/-α-2b	48/77 (38.4/61.6)
Dose of ribavirin (mg/day)	867 (800-1000)

Abbreivations: ALK-p: alkaline phosphatase; ALT: alanine aminotransferase; AST: aspartate aminotransferase; BMI: body mass index; Hgb: hemoglobin; HCV: hepatitic C virus; PT INR: international normalized ratio of prothrombin time; WBC: white blood cells; Peg-IFN: pegylated-interferon.

^**#**^Only the patients undergoing liver biopsy were analyzed for fibrosis and necroinflammatory activity of liver tissue. F1-F4 represent fibrotic scores, and A0-A3 mean necroinflammatory scores, according to METAVIR scoring system [10].

^**§**^One patient had undergone splenectomy due to traumatic splenic rupture.

After 24-week follow-up of platelet counts, 46 patients (40.4%) were classified as having improved platelet counts, and 54 patients (47.4%) stationary platelet counts. Only 14 patients (12.3%) had a decline in platelet count. The maximal decline in platelet count was 32% ± 22% (mean ± standard deviation). A total of 16 patients (12.8%) had severe thrombocytopenia necessitating dose reductions, while 32 patients (25.6%) had mild thrombocytopenia, and 77 patients (61.6%) had moderate thrombocytopenia. No mortality or morbidity necessitating hospitalization owing to severe thrombocytopenia was encountered in the 16 patients. Five patients (4%) discontinued treatment due to thrombocytopenia. These patients had lower albumin levels (3.8 ± 0.2 mg/dL vs. 4.2 ± 0.3 mg/dL, *P *= 0.014), lower baseline platelet counts (94,400 ± 29,399/μL vs. 122,867 ± 19,843/μL, *P *= 0.035), and higher international normalized ratio (INR) of prothrombin time (PT) (1.14 ± 0.06 vs. 1.05 ± 0.07, *P *= 0.008) than did the other 120 patients, who did not discontinue treatment because of severe thrombocytopenia. These five patients also had higher bilirubin (1.1 ± 0.3 mg/dL vs. 0.8 ± 0.3 mg/dL, *P *= 0.066) and alkaline phosphatase levels (129 ± 50 U/L vs. 97 ± 31 U/L, *P *= 0.099), and a greater proportion of them had splenomegaly (60% vs. 20.2%, *P *= 0.068), a difference of borderline significance. There was a trend of toward discontinuing therapy due to severe thrombocytopenia in patients with cirrhosis (40% vs. 21.7% in patients without cirrhosis %, *P *= 0.312); however, this difference did not reach statistical significance. Nevertheless, the rates of SVR were comparable between patients with and without severe thrombocytopenia (60.0% vs. 68.7%, *P *= 0.728) during antiviral therapy.

Low body mass index (*P *= 0.022), low albumin (*P *= 0.020), low baseline HCV-RNA level (*P *= 0.033), genotype 2 (*P *= 0.033), low baseline platelet count (*P *< 0.001), and high rate of platelet decline at week 2 (i.e., early platelet decline, *P *< 0.001) were significantly correlated with the development of severe thrombocytopenia by univariate analysis (Table 2). In addition, higher alkaline phosphatase level (*P *= 0.065) and lower mean ribavirin dose (*P *= 0.079) were borderline significant. The cut-off values with best accuracy of continuous variables among these factors were identified by ROC analysis. The cut-off value of baseline platelet count was 98,500/μL, while that of early platelet decline was 30.51%. A baseline platelet count of 100,000/μL, and early platelet decline of 30%, were used for simplicity. Multivariate logistic regression (Table 3) revealed that only baseline platelet count < 100,000/μL (*P *< 0.001, odds ratio [OR] 179.219; 95% C.I., 12.219-2628.602), and early platelet decline > 30% (i.e., rapid early platelet decline, *P *= 0.003, OR, 45.742; 95% C.I., 3.524-593.689) were significantly associated with development of severe thrombocytopenia. The positive and negative predict values (i.e. PPV and NPV) of baseline platelet count < 100,000/μL were 50% and 95.1%, and those of rapid early platelet decline were 35.1% and 96.6%, respectively. The PPV and NPV of a combination of these two factors were 100% and 97.4%, respectively. In the subgroup of patients with baseline moderate thrombocytopenia, rapid early platelet decline has not only high PPV (100%), but also high NPV (91.7%).

**Table 2:** Significant factors related to severe thrombocytopenia by univariate analysis

	Univariate analysis		
	
Factors	Platelets < 50 × 10^3^/μL	**Platelets ≥ 50 × 10**^**3**^**/μL**	*P *value
Age, years	58 ± 11	57 ± 10	0.663
Male/female	9/7	56/53	0.463
BMI, kg/m^2^	23.37 ± 2.65	25.30 ± 3.19	0.022
ALB, g/dL	3.96 ± 0.30	4.16 ± 0.30	0.020
Total bilirubin, mg/dL	0.9 ± 0.4	0.8 ± 0.3	0.179
AST, U/L	147.44 ± 80.26	116.78 ± 62.33	0.145
ALT, U/L	200 ± 130	196 ± 116	0.909
ALK-p, U/L	113.20 ± 32.52	96.56 ± 31.62	0.065
Prothrombin time (INR)	1.07 ± 0.07	1.05 ± 0.05	0.111
WBC count,/cumm	4954 ± 1169	5303 ± 1215	0.338
Hgb, g/dL	13.86 ± 0.86	14.47 ± 1.49	0.101
Platelets, × 10^3^/μL	100.63 ± 23.21	124.83 ± 18.74	< 0.001
Necroinflammatory activity, A0+A1/A2+A3	6/5	56/19	0.152
Fibrosis status, F1+F2/F3+F4	4/8	29/50	0.547
Cirrhosis, yes/no	5/11	23/86	0.269
Splenomegaly yes/no (%)	5/11	22/86	0.247
HCV-RNA, log_10 _IU/mL	5.41 ± 1.42	6.09 ± 0.96	0.033
HCV genotype- 1/2	5/11	66/43	0.033
Peg-IFN-α-2a/-α-2b	6/10	42/67	0.583
Peg-IFN-α-2a dose, μg/week	180 ± 0 (n = 6)	179 ± 9 (n = 42)	0.705
Peg-IFN-α-2b dose, μg/week	89 ± 11 (n = 10)	93 ± 12 (n = 67)	0.370
Ribavirin dose, mg/day	811 ± 169	900 ± 166	0.079
Rate of PLT decline at week 2, %	38.46 ± 11.53	14.06 ± 21.85	< 0.001

Abbreviations: ALB: albumin; ALK-p: alkaline phosphatase; AST: aspartate aminotransferase; BMI: body mass index; Hgb: hemoglobin; HCV: hepatitic C virus; Peg-IFN: peginterferon; PLT: platelet

**Table 3:** Multivariate analysis of factors related to severe thrombocytopenia

	Multivariate Analysis		
	
Factors	*P *value	Odds Ratio	95% Confidence Interval
BMI (< 24.5/≥ 24.5 Kg/m^2^)	0.280		
ALB (≤ 4.1/> 4.1 g/dL)	0.412		
ALK-p (≥ 97/< 97 U/L)	0.646		
PLT (< 100,000/≥ 100,000/μL)	< 0.001	179.219	12.219-2628.602
HCV-RNA (log_10_, IU/mL) (≥ 5.95/< 5.95)	0.196		
HCV genotype (1/2)	0.419		
Ribavirin dose (≤ 24.58/> 24.58)	0.201		
Rate of PLT decline at week 2 (early PLT decline, > 30%/≤ 30%)	0.003	45.742	3.524-593.689

Abbreviations: ALB: albumin; ALK-p: alkaline phosphatase; AST: aspartate aminotransferase; PLT: platelet; BMI: body mass index; C.I.: confidence interval; HCV: hepatitic C virus.

## Discussion

Thrombocytopenia, encountered frequently among patients with HCV-related chronic hepatitis and cirrhosis, is usually aggravated during IFN-based antiviral therapy. In several large-scale clinical trials, the incidence of severe on-treatment thrombocytopenia was 3-5% among all patients with chronic hepatitis C [2-4]. In the current study, severe thrombocytopenia occurred more frequently (12.8%) in patients with baseline thrombocytopenia. Roomer et al. (2010) documented that patients with baseline thrombocytopenia were vulnerable to severe thrombocytopenia [5]. This group also found that severe thrombocytopenia was significantly associated with bleeding events. In the present study, severe thrombocytopenia did occur in some patients during antiviral therapy, but it was not followed by mortality or major morbidity. Instead, severe thrombocytopenia was associated with higher rates of premature discontinuation of therapy.

Thrombocytopenia is one of the extrahepatic manifestations of HCV infection. Several mechanisms have been proposed to account for thrombocytopenia in patients with CHC [12-15]. Hypersplenism in the cirrhosis stage, autoantibodies or immune complexes directed against platelets, direct infection of platelets and megakaryocytes by HCV, decreased level or activity of thrombopoietin (TPO), and virus-induced bone marrow suppression or direct platelet suppression are linked to the low platelet counts in HCV-infected patients before antiviral therapy [12-27]. In the present study, 22% of patients with baseline thrombocytopenia had splenomegaly. In these patients, hypersplenism might account for thrombocytopenia; however, the other 78% of patients with baseline thrombocytopenia did not have splenomegaly; other causes such as decreased level or activity of TPO, autoimmune reaction to platelets, and direct infection of platelets and megakaryocytes by HCV might contribute to the thrombocytopenia.

The proposed mechanisms of treatment-related thrombocytopenia include inhibition of proliferation of megakaryocytes from IFN-α [28]; and, less commonly, autoimmune reactions [29] and impaired TPO production [30]. Direct repression of megakaryopoiesis by IFN-α by inhibiting TPO-induced signaling was demonstrated in one *in vitro *study [31]. In contrast, ribavirin also plays a role in thrombocytopenia because ribavirin is associated with reactive thrombocytosis [3]. Determinants of severity of thrombocytopenia during antiviral therapy may be numerous, and the interaction between these factors may be complicated. Detection of some factors, such as TPO and anti-platelet antibodies, is rarely conducted clinically, and is not practical. Hence, the authors of the current study hypothesized that early platelet dynamics, which could represent the summation of a variety of factors, was a relevant factor. Among the limited reports, the search for predisposing factors for severe thrombocytopenia during antiviral therapy disclosed discrepancies between these studies. For example, Roomer et al. (2010) found baseline thrombocytopenia and cirrhosis [5], whereas Nachnani et al. (2010) reported that lower white blood cell counts, higher alkaline phosphatase, and higher iron level, were associated with severe thrombocytopenia during antiviral therapy [32]. The present study was aimed at finding factors associated with severe thrombocytopenia in patients with baseline thrombocytopenia, and observed that baseline platelet counts < 100,000/μL and rapid early platelet decline (> 30% decline of platelets 2 weeks after initiation of therapy) were strong factors for predicting the development of severe thrombocytopenia during antiviral therapy. Lower baseline platelet counts, which reportedly correlate with decreased liver function and lower TPO level [17], account for lower on-treatment platelet counts. Alternatively, rapid early platelet decline may represent higher repression of megakaryopoiesis, or increased platelet consumption. Further studies assessing megakaryocytes, autoimmune anti-platelet antibodies, and virological response during combination therapy are warranted to elucidate factors associated with rapid early platelet decline, and consequent development of severe thrombocytopenia.

The other factor, cirrhosis, reported by Roomer et al. (2010) as a significant factor in severe thrombocytopenia during antiviral therapy in the general population with chronic hepatitis C, was not relevant in the current study. Although low pretreatment platelet count and albumin level, suggestive of cirrhosis status, were significant on univariate analysis, the significance of cirrhosis in prediction of on-treatment severe thrombocytopenia may be restricted by small sample size. In addition, the majority of patients without cirrhosis in the present study were classified as having advanced hepatic fibrosis (F3). It is reasonable to postulate that the increased susceptibility to thrombocytopenia in these patients may also decrease the difference in the rate of severe thrombocytopenia in patients with cirrhosis compared with the rate in those without cirrhosis.

In patients with pretreatment thrombocytopenia, it is possible to select a group at low risk for severe on-treatment thrombocytopenia by means of baseline platelet count before antiviral therapy. For pretreatment platelet count ≥ 100,000/μL, the NPV for severe on-treatment thrombocytopenia was 95.1%, i.e., the on-treatment incidence of severe thrombocytopenia in the patients with baseline platelet count ≥ 100,000/μL was only 4.9%, which is comparable to that of the general population with chronic hepatitis C during antiviral therapy (which was reportedly 3-5%). In high-risk patients, it is still possible to identify those with a relatively low-risk (8.3%) of severe thrombocytopenia by lack of rapid early platelet decline (11 among 12 (91.7%) patients with baseline moderate thrombocytopenia and platelet decline ≤ 30%).

In contrast, patients with platelet count < 100,000/μL before treatment and a rapid early platelet decline during antiviral therapy were at very high risk (100%) for severe thrombocytopenia (positive predictive value was 100%). For this group of patients, modification of antiviral therapy, close monitoring, and salvage therapy for thrombocytopenia were needed. A TPO-mimetic agent may be beneficial in these high-risk patients. An emerging TPO-mimetic agent, eltrombopag, reportedly increases the proportion of baseline-thrombocytopenic, HCV-infected patients completing the initial 12 weeks of antiviral therapy, from 36% to 65% (compared with 6% in the control group) in a phase II study [6]. Eltrombopag is not yet licensed for treatment of thrombocytopenia in patients with chronic liver disease because the risk of portal venous thrombosis (PVT) increased substantially when this drug was administered at 75 mg daily for 14 days [33]. Subsequent PVT had never been reported in patients undergoing combination therapy with peg-IFN-α and ribavirin, except in those having undergone splenectomy or partial splenic embolization prior to treatment [34,35]. Hence, eltrombopag was believed to be responsible for PVT, and such conditions were different from those assessed in the current study. Nevertheless, cautious use of this drug at a low dose was recommended in concert with close monitoring of patients [33]. Still under investigation is the use of other TPO-mimetic agents, including romiplostim (which has been approved for treatment of immunological thrombocytopenic purpura) in patients with chronic liver disease and thrombocytopenia,.

The study presented here has several limitations. First, the choice between peg-IFN-α-2a and peg-IFN-α-2b was not randomized; however, this study demonstrated that the choice of peg-IFN-α-2a or peg-IFN-α-2b was not an independent factor related to severe thrombocytopenia. A meta-analysis study conducted by Alavian SM et al. also failed to find significantly different rates of thrombocytopenia (platelet count < 50,000/μL) between both types of peg-IFN-α (odds ratio 1.37, 95% CI 0.73-2.58) [36]. Second, the number of patients with baseline thrombocytopenia was relatively small; therefore, factors not demonstrated to be significant in this study may become significant in larger studies. Third, due to the retrospective nature of our study, further prospective investigations are needed.

## Conclusions

Severe thrombocytopenia (platelet counts < 50,000/μL), encountered by a substantial portion (12.8%) of patients with baseline thrombocytopenia, was associated with a higher rate of premature cessation of therapy. In these thrombocytopenic patients, pretreatment platelet levels < 100,000/μL and rapid early platelet decline (> 30% decline of platelets after 2 weeks of therapy) were strongly associated with the occurrence of treatment-related severe thrombocytopenia. Patients with pretreatment thrombocytopenia should be informed early about the potential for development of severe thrombocytopenia, should be monitored closely during therapy, and, potentially, should be administered TPO-mimetic agents.

## List of abbreviations

CHC: Chronic hepatitis C; peg-IFN-α: pegylated interferon-α; SVR: sustained virological response; HCV: hepatitis C virus; ALT: alanine aminotransferase; ULN: upper limit of normal; RVR: rapid virologic response; ANC: absolute neutrophil count; Hgb: hemoglobin; OR: odds ratio; NPV: negative predictive value; PPV: positive predictive value; TPO: thrombopoietin.

## Competing interests

The authors declare that they have no competing interests.

## Authors' contributions

KHLi and HCY contributed to study concept, study design, data acquisition, statistical analysis, data interpretation, and writing of the manuscript. CKL, WLT, and WCC contributed to data acquisition, data interpretation, and manuscript preparation. PIH and KHLa contributed to study design, and critical revision of the manuscript. HHC contributed to the revised manuscript. All authors read and approved the final manuscript.

## Pre-publication history

The pre-publication history for this paper can be accessed here:

http://www.biomedcentral.com/1471-230X/12/7/prepub
